# A method to analyze the sensitivity ranking of various abiotic factors to acoustic densities of fishery resources in the surface mixed layer and bottom cold water layer of the coastal area of low latitude: a case study in the northern South China Sea

**DOI:** 10.1038/s41598-020-67387-7

**Published:** 2020-07-07

**Authors:** Mingshuai Sun, Yancong Cai, Kui Zhang, Xianyong Zhao, Zuozhi Chen

**Affiliations:** 1 South China Sea Fisheries Research Institute, Chinese Academy of Fishery Sciences, Guangzhou, 510300 China; 2 Key Laboratory of Open-Sea Fishery Development, Ministry of Agriculture and Rural Affairs, Guangzhou, 510300 China; 3 Southern Marine Science and Engineering Guangdong Laboratory, Guangzhou, 511458 China; 4 Shanghai Ocean University, Shanghai, 200120 China; 5 Yellow Sea Fisheries Research Institute, Chinese Academy of Fishery Sciences, Qingdao, 266237 China

**Keywords:** Behavioural ecology, Biogeography, Biogeography

## Abstract

This is an exploratory analysis combining artificial intelligence algorithms, fishery acoustics technology, and a variety of abiotic factors in low-latitude coastal waters. This approach can be used to analyze the sensitivity level between the acoustic density of fishery resources and various abiotic factors in the surface mixed layer (the water layer above the constant thermocline) and the bottom cold water layer (the water layer below the constant thermocline). The fishery acoustic technology is used to obtain the acoustic density of fishery resources in each water layer, which is characterized by Nautical Area Scattering Coefficient values (NASC), and the artificial intelligence algorithm is used to rank the sensitivity of various abiotic factors and NASC values of two water layers, and the grades are classified according to the cumulative contribution percentage. We found that stratified or multidimensional analysis of the sensitivity of abiotic factors is necessary. One factor could have different levels of sensitivity in different water layers, such as temperature, nitrite, water depth, and salinity. Besides, eXtreme Gradient Boosting and random forests models performed better than the linear regression model, with 0.2 to 0.4 greater R^2^ value. The performance of the models had smaller fluctuations with a larger sample size.

## Introduction

The thermocline in the low latitude sea area is permanent, the upper layer of the thermocline is a surface mixed layer, and the lower layer of the thermocline is the bottom cold water layer. The distribution of fishery organisms and their driving factors in the perfusing water layers (especially the surface mixed layer and the bottom cold water layer) deserves further investigation. The offshore of the northern South China Sea is a typical representative of low-latitude coastal waters, and it is also an important traditional fishery production operation in China, owing to its good climate that offers a conducive habitat for the marine life, a spawning site, and a place for fattening and farming of fish. However, fishery resources have increasingly become small and of reduced quality^1,2^. Resource density, the single yield of fishing vessels, and catch quality are declining. The catch rates of most economical fish have fallen to very low levels. High-quality commercial fish are facing depletion. The decline of fishery resources is more serious in coastal waters^3^. These phenomena could be related to probability distribution characteristics, abiotic factors, and overfishing^1,4^.

Fishery acoustics has emerged as an important modality for investigating and evaluating marine life. It is superior to traditional bottom trawling, as these techniques are more direct and effective and generate abundant data, especially in the case of large distances among sites and a small number of samples. Nautical area scattering coefficient (NASC) refers to the sum of coefficients from all species in the profile data^5^. It indicates the probability distribution characteristics of fishery resources^5–9^. Previous studies have analyzed only the overall water layer, and none of them carried out a detailed analysis of the water layers. The fishery acoustic technology, like a scalpel, cuts the entire body of water into multiple layers for further research. We compared the data obtained for the same duration among different water layers.

In addition, it is always challenging in marine surveys to decide over the number and density of survey sites and the number of samples to be collected. It is difficult to conduct investigations in the northern coastal waters of the South China Sea due to its large extent. Usually, the distance between the sites is extremely large, which hinders the analysis of the sensitivity of abiotic factors in a multitude of water conditions.

Another problem that has to be faced is the analysis method of multi-feature datasets, such as the 41 abiotic factors used in this study. The models available today are linear models (including generalized linear models), additive models (including generalized additive models), and complex models (including ensemble learning, deep learning, and others). In general, models vary greatly in their expressiveness (formulaic or graphical expression) and accuracy. For example, the linear model has the best expressiveness, but its accuracy is the worst. The additive model is less expressive than the linear model, but it is more accurate. The accuracy of the complex model is the highest, but the expression ability is poor. In addition, the generalized additive model assumes that the independent variables are not related to each other in order to improve accuracy. Complex models can achieve high accuracy without considering the correlation between independent variables. Deep learning often requires a large amount of data. In integrated learning, two algorithms stand out, XGBoost and random forests, which can not only achieve high accuracy but also do not need a large number of samples. XGBoost (eXtreme Gradient Boosting)^10^ is also known as a gradient boosting algorithm. It is a machine learning technique for regression and classification problems, and it is faster than other algorithms. Random forests algorithm is an extension of bagging^11^. XGBoost and random forests are widely used in several areas, such as image classification^12,13^, data analysis^14–16^, and information classification^17,18^. They are also used to evaluate the sensitivity of features and calculate the sensitivity scores of all abiotic factors^19,20^.

In order to address these problems and meet the aforementioned requirements, we considered the short-term data of a voyage as the research object and conducted this exploratory study. At the same time, we also hope that this idea of combining the artificial intelligence algorithm with professional fields and conducting multidimensional data analysis can inspire researchers in other fields.

## Materials and methods

### Site description and sampling

**Figure 1:**
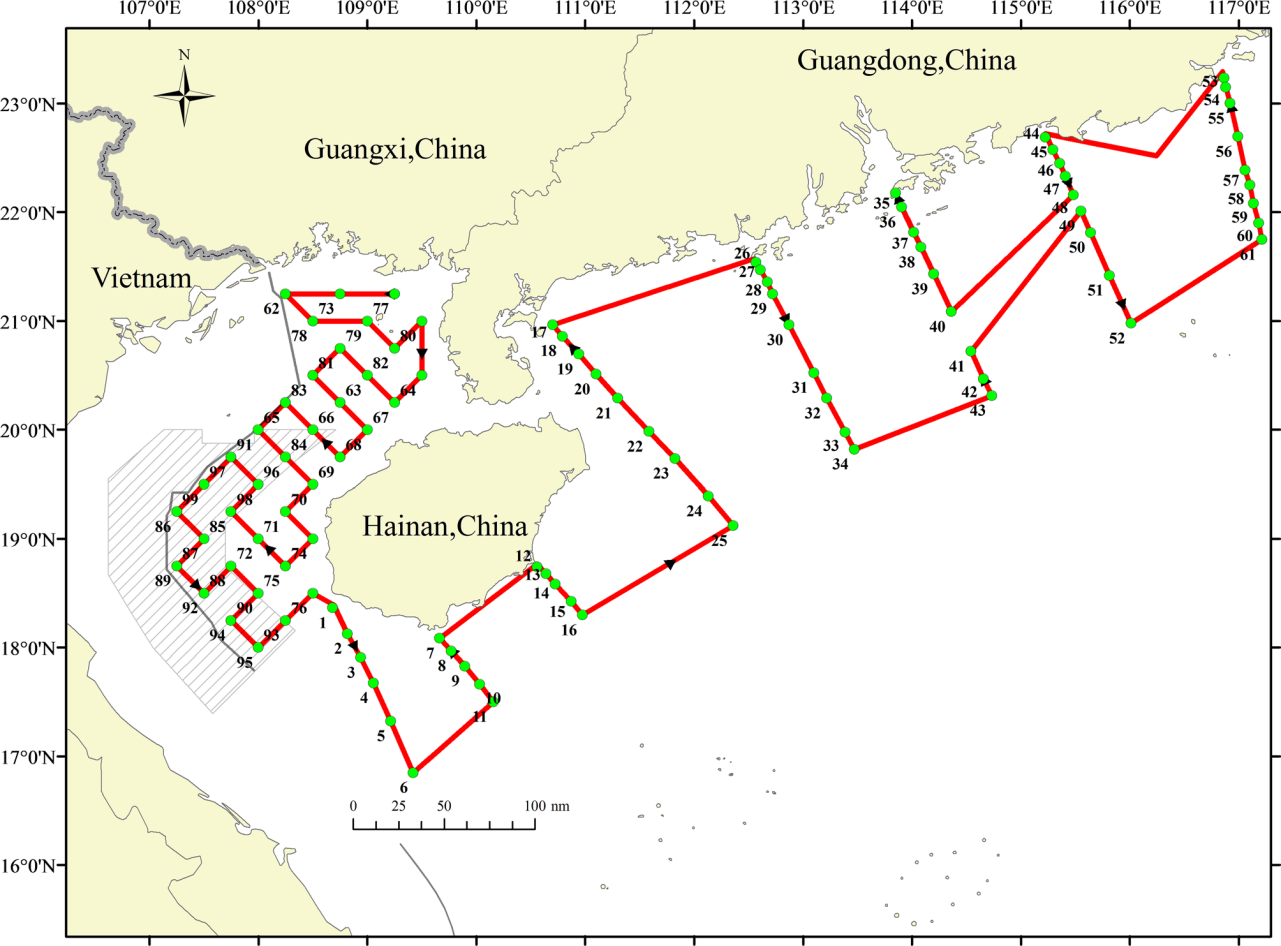
Acoustic navigation route (red lines) and sampling sites (green points ) employed during the fishery. Map created in ArcGIS Desktop 9.3. https://www.esrichina.com.cn/.

Acoustic data were collected using single boat bottom otter trawl (engine: 441 kW, gross tonnage: 242 t, length of boat: 36.8 m, width: 6.8 m) in the offshore of the Northern South China Sea, named as north fishing 60011, with a scientific fisheries portable echo sounder (70 kHz and 120 kHz; Fig. 1).

Fishery samples were collected from 99 sites by single boat bottom otter trawls, with 404 type otter trawl, 80.80 m circumference, and 20 cm mesh size around the leading edge of the net. The total length of the net was 60.54 m and the mesh size of the codend was 39 mm. It took 60 min per site. Then, the sum mass and the number of samples were measured.

Water temperature, salinity, and depth of water were obtained by AML Plus X, and other indexes were also collected, such as nutrients and transparency. The sampling depths for nutrients were 0 m, 10 m, and 20 m.

### Data preprocessing

Echoview software (Version 6.1, https://www.echoview.com/) was used for the analysis of acoustic data. All data were checked carefully, and data not from the routes were excluded. Data from two water layers were analyzed in the surface mixed layer (20 m below effective acoustic data line, except for blind zone) and the bottom cold water layer (20 m above effective acoustic data line, except for blind zone). The basic integral voyage unit selected was 5 nmi. The integral threshold was set as − 80 dB. Background noise was removed, and surface and bottom NASC (m^2^/nmi^2^) integral values were collected, which were also fishery density for the same volume, as the sampling range for surface and bottom cold water layers was the same.

The samples collected were divided into two categories based on their living area: surface mixed layer (the sediment surface and some subsurface layers) and bottom cold water layers. Cephalopods live in the bottom cold water layer during the day and leave the bottom during the night. Therefore, the number of Cephalopods was counted in the two layers with a ratio of 0.5:0.5.

Abiotic factors contain both primary and derived factors. Primary features include surface salinity (SS, ppt) and surface temperature (ST, °C) at 2 m in the surface mixed layer, bottom salinity (BS, ppt) and bottom temperature (BT, °C) at 2 m in the bottom cold water layer, water depth (WD, m), longitude (X, °), latitude (Y, °), transparency (TRA, m), and chlorophyll concentration (CHL, mg/m^3^). The derived factors calculated on the basis of primary factors included salinity difference (DS, ppt) and temperature difference (DT, °C) between the surface and bottom cold water layers, concentration difference between NO_2_^−^ at 0 m and 10 m (N2-d010, mg/L), and few others as shown in Table 1.

**Table 1:** List and grouping of abiotic factors.

Group	Factor (abbreviation)	Factor (note)	Unit	Remark
Geographic factors	WD	Water depth	m	
	X	Longitude	°	
	Y	Latitude	°	
Static factors	SS	Surface salinity	ppt	Surface
	BS	Bottom salinity	ppt	Bottom
	ST	Surface temperature	°C	Surface
	BT	Bottom temperature	°C	Bottom
	TRA	Transparency	m	Surface
	CHL	Chlorophyll concentration	mg/m^3^	Surface
	N2-0 m	NO_2_^−^ 0 m concentration	mg/L	Surface
	N2-10 m	NO_2_^−^ 10 m concentration	mg/L	Surface
	N2-20 m	NO_2_^−^ 20 m concentration	mg/L	Bottom
	N3-0 m	NO_3_^−^ 0 m concentration	mg/L	Surface
	N3-10 m	NO_3_^−^ 10 m concentration	mg/L	Surface
	N3-20 m	NO_3_^−^ 20 m concentration	mg/L	Bottom
	N4-0 m	NH_4_^+^ 0 m concentration	mg/L	Surface
	N4-10 m	NH_4_^+^ 10 m concentration	mg/L	Surface
	N4-20 m	NH_4_^+^ 20 m concentration	mg/L	Bottom
	P-0 m	PO_4_^3−^ 0 m concentration	mg/L	Surface
	P-10 m	PO_4_^3−^ 10 m concentration	mg/L	Surface
	P-20 m	PO_4_^3−^ 20 m concentration	mg/L	Bottom
	Si-0 m	SiO_3_^2−^ 0 m concentration	mg/L	Surface
	Si-10 m	SiO_3_^2−^ 10 m concentration	mg/L	Surface
	Si-20 m	SiO_3_^2−^ 20 m concentration	mg/L	Bottom
Dynamic factors	DS	Salinity difference between surface and bottom layers	ppt	
	DT	Temperature difference between surface and bottom layers	°C	
	N2-d010	Concentration difference between NO_2_^−^ 0 m and 10 m	mg/L	
	N2-d020	Concentration difference between NO_2_^−^ 0 m and 20 m	mg/L	
	N2-d1020	Concentration difference between NO_2_^−^ 10 m and 20 m	mg/L	
	N3-d010	concentration difference between NO_3_^−^ 0 m and 10 m	mg/L	
	N3-d020	Concentration difference between NO_3_^−^ 0 m and 20 m	mg/L	
	N3-d1020	Concentration difference between NO_3_^−^ 10 m and 20 m	mg/L	
	N4-d010	Concentration difference between NH_4_^+^ 0 m and 10 m	mg/L	
	N4-d020	Concentration difference between NH_4_^+^ 0 m and 20 m	mg/L	
	N4-d1020	Concentration difference between NH_4_^+^ 10 m and 20 m	mg/L	
	P-d010	Concentration difference between PO_4_^3−^ 0 m and 10 m	mg/L	
	P-d020	Concentration difference between PO_4_^3−^ 0 m and 20 m	mg/L	
	P-d1020	Concentration difference between PO_4_^3−^ 10 m and 20 m	mg/L	
	Si-d010	Concentration difference between SiO_3_^2−^ 0 m and 10 m	mg/L	
	Si-d020	Concentration difference between SiO_3_^2−^ 0 m and 20 m	mg/L	
	Si-d1020	Concentration difference between SiO_3_^2−^ 10 m and 20 m	mg/L	

The present study classified all factors into three groups: (1) geographic factors, containing water depth (WD, m), longitude (X, °) and latitude (Y, °); (2) dynamic factors, with all derived factor (total 17), and (3) other 21 miscellaneous factors such as surface and bottom factors, belonging to static factors. Transparency (TRA, m) and chlorophyll concentration (CHL, mg/m^3^) were defined as surface static characteristics, and abiotic factors at 20 m were defined as bottom static characteristics. Therefore, in the present study, surface factors reflected surface static characteristics and bottom factors represented bottom static characteristics. See Table 1 below for details.

### Data expansion and random sampling

The sample size, as an important part of the analysis on abiotic factors, was less than 100 in the offshore of the northern South China Sea in this study, which was limited by the number of survey sites. Therefore, it was not enough to comprehensively analyze the data. However, we collected coordinate information for every sample so that surface data could be obtained based on interpolation methods, and subsequently, random sampling from surface data was performed, and the effect of analytical models with different sample sizes was evaluated. Interpolation methods included Kriging interpolation and inverse distance weighting (IDW). The methods were selected on the basis of the highest goodness of fit (*R*^2^) and minimum mean square error (MSE). The size of random sampling was set as 100, 200, 300, 400, 500, and 600. In order to avoid over-concentration in sampling, we set the minimum sampling interval. The spacing of sampling points affects the number of sampling points and the degree of sampling dispersion. The randomness of sampling is also considered. So, after many attempts, we set the minimum interval between the sites to 5 nautical miles. However, 2,100 samples, the sum of all sites, were not restricted to the distance.

### Data modeling

The relationships between the NASC and 41 abiotic factors were determined with XGBoost, random forests, and linear regression models. Furthermore, all the 41 abiotic factors sampled were dimensionless through standardization or Z-score normalization. The model effect was estimated on the basis of the highest goodness of fit (*R*^2^) and MSE from cross-validation methods. The proportion between the training dataset and the testing dataset was 7:3. According to Zhou^21^, when the amount of data is small, about 2/3 to 4/5 of the sample data will be used for training, and the rest will be used for testing. Besides, 7:3 of the training data and test data are also a kind of allocation ratio usually employed for small data, which can effectively improve the generalization ability of the model.

Both XGBoost and random forests models are based on multiple decision trees on the same dataset. Random forests model generates several trees, and each is independent^11^ with leaves of equal weight within the model for obtaining higher accuracy. XGBoost introduces leaf weighting to penalize those that do not improve the model predictability^10^. In order to improve the efficiency of model optimization, some important parameters can be selected to adjust. If satisfactory results have been achieved, model optimization can be ended. If the researchers are not satisfied with the results of model optimization, they can choose more complex parameters for deeper adjustment. Here, we only made adjustments to certain parameters, such as the learning_rate, n_estimators, and the subsample^22^. With the optimized model, feature weighting of the 41 factors on surface and bottom fishery resources density was calculated by XGBoost and random forests models, and their sensitivity scores were obtained. Thus, the descending analysis was performed.

The analysis was performed in Python 3.7 using the Scikit-Learn package^23^ and the XGBoost library^10,22^.

### Sensitivity of factors

Generally, the analysis of the sensitivity of factors provides a score that indicates the value of each feature in the construction of decision trees within the model. To avoid the occurrence of errors, the average scores from XGBoost and random forests models were calculated for major factors affecting the water quality. The sum contribution was set as 50%, 80%, and 95%, ranked from level one to level four, meaning the first level was the highest, and the fourth was the lowest.

### Comparison of factors between the surface and bottom cold water layers

The difference and sum of each factor between the surface and bottom cold water layers were obtained and ranked. Moreover, comparisons of the factors between these two water layers were made. The rank of difference represents the sensitivity value of each factor in different water layers, and the sum indicates the overall role of factors. All factors were divided into three categories: Group A, factors are important for surface and not for the bottom cold water layer, and the difference value is greater than 0.05. Group B factors are important for the bottom and not for the surface mixed layer, and the difference value is smaller than 0.05. Group C factors are important for both surface and bottom cold water layers, and the difference value is around 0. It is defined as C + with the sum value greater than 0.05, meaning that these factors are important for two water layers. On the contrary, it will be treated as C − , with less sensitivity for two water layers.

The difference in the importance of each factor between the surface layer and the bottom layer was calculated and sorted according to the difference value. Similarly, the sum of the importance of each factor between the surface layer and the bottom layer was calculated and sorted according to the sum value. Factors can be divided into three categories: Class A, which is of high importance for the surface layer, but very low for the bottom layer, difference value >  + 0.05; Class B, which is more important for the bottom, but less important for the surface layer, difference value < – 0.05; Class C, the difference value is approximately the same for the bottom layer as for the surface layer, difference value ≈ 0. In addition, in Class C, when the sum value is greater than 0.05, it is defined as C + , which means that such factors are more important for both the surface layer and the bottom layer. When the sum value is less than 0.05, it is defined as C–, which means that such factors are of only slight importance to the surface and bottom layers.

## Results

### Probability distribution characteristics of surface and bottom fishery resources and compositions of catches

**Figure 2:**
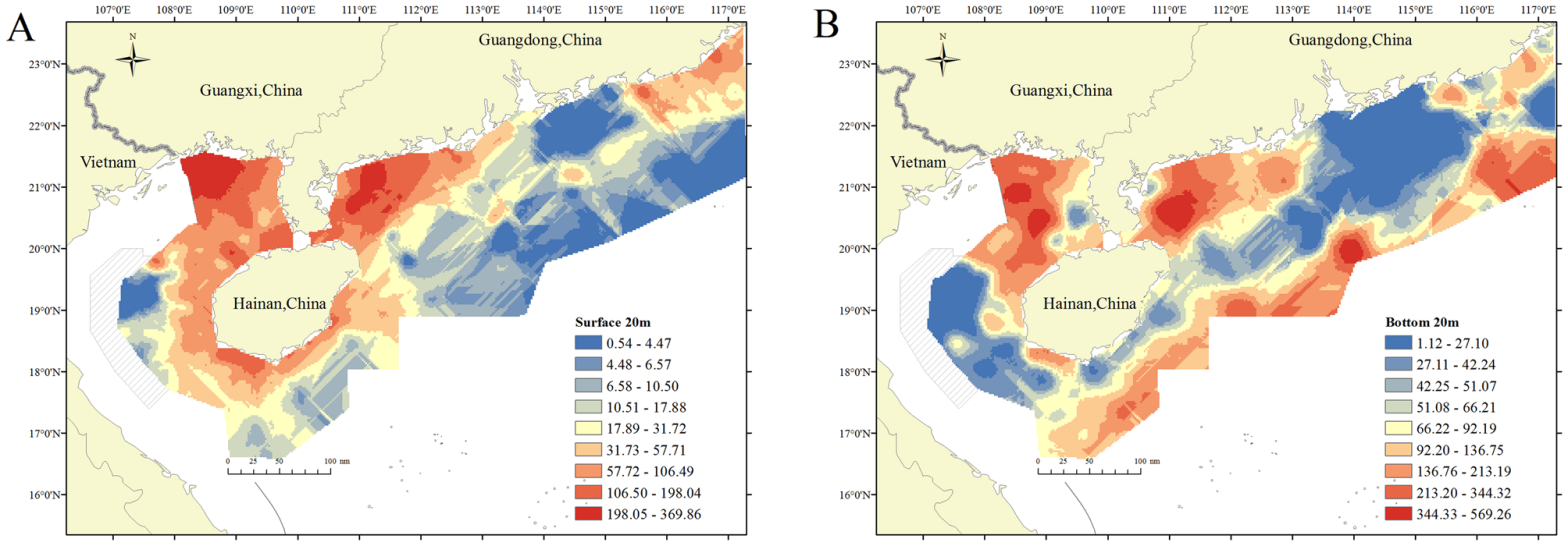
The distribution characteristics of nautical area scattering coefficients (NASC) in the surface mixed layer (**A**) and the bottom cold water layer (**B**) at a depth of 20 m based on geometrical interval classification. The average value is 43.39, and the standard deviation is 54.73 for panel (**A**), and the average value is 106.00, and the standard deviation is 91.02 for panel (**B**). Map created in ArcGIS Desktop 9.3. https://www.esrichina.com.cn/.

Surface NASC mainly concentrated around the Hainan island, and the bottom NASC was concentrated north of Hainan Island and in the southwestern waters of Guangdong Province (above 100 m). The bottom NASC was twice greater than the surface NASC (Fig. 2). Fish species were rich (Table 2), and the weight ratio and the density ratio in terms of the number of species captured in bottom and surface mixed layers were 2.13 and 1.94 (Table 3), respectively, close to the ratio of the bottom NASC to the surface NASC.

**Table 2:** Class statistics of catches, biomass, and individual number.

Fish species	Habitat layer	Weight(kg)	Number
*Pneumatophorus japonicus, Rastrelliger kanagurta*	Surface layer	17.166	165
*Ariomma indica, Psenopsis anomala*	Surface layer	603.075	8,292
*Decapterus maruadsi*	Surface layer	774.339	23,597
*Trachurus japonicas*	Surface layer	937.429	25,117
*Sardinella aurita, Sardinella jussieu*	Surface layer	290.454	23,600
*Tentoriceps cristatus, Trichiurus haumela, Trichiurus nanhaiensis, Trichiurus brevis*	Near-bottom layer	237.824	3,399
*Navodon xanthopterus*	Near-bottom layer	276.389	13,139
*Argyrosomus aneus, Argyrosomus macrocephalus, Argyrosomus pawak, Argyrosomus argentatus*	Near-bottom layer	151.949	9,170
*Saurida undosquamis, Saurida tumbil, Saurida elongata*	Near-bottom layer	206.032	2,786
*Evynnis cardinalis*	Near-bottom layer	784.218	26,980
*Priacanthus macracanthus, Priacanthus tayenus*	Near-bottom layer	269.555	8,107
*Nemipterus virgatus, Nemipterus bathybius, Nemipterus oveni, Nemipterus japonicus, Nemipterus nemurus*	Near-bottom layer	412.087	9,362
*Upeneus bensasi, Upeneus sulfureus, Upeneus moluccensis, Parupeneus chrysopleuron*	Near-bottom layer	116.327	4,250
*Siganus oramin, Siganus fuscescens*	Near-bottom layer	66.004	7,241
*Acropoma japonicum, Acropoma hanedai*	Near-bottom layer	414.008	8,343
*Loligo edulis*	Near-bottom layer during the day, Surface layer at night	233.187	6,234
*Loligo chinensis*	Near-bottom layer during the day, Surface layer at night	43.991	1,339
Other cephalopods	Near-bottom layer during the day, Surface layer at night	1,280.103	269,370
Other species	Substratum species are the majority	3,524.761	195,051

**Table 3:** Statistics of catches in the different water layers.

Habitat layer	Catch weight (kg)	Catch number	Weight %	Number %
Surface	3,401.10	219,243	0.32	0.34
Near-bottom	7,237.79	426,299	0.68	0.66

Other cephalopoda species mainly include: *Loligo beka, Loligo duvaucelii, Loligo tagoi*, *Sepioteuthis lessoniana*, *Sepia esculenta, Sepia latimanus, Sepia lycidas, Sepia pharaonis*, *Sepiella maindroni*, *Metasepia tullbergi and Euprymna berryi.* Other species evaluated are: *Raja hollandi, Dasyatis zugei, Trachinocephalus myops, Rhynchocymba nystromi, Muraenesox cinereus, Fistularia petimba, Sphyraena pinguis, Epinephelus sexfasciatus, Apogonichthys ellioti, Branchiostegus argentatus, Leiognathus ruconius*, *Therapon theraps, Pampus chinensis, Pterois lunulata, Lepidotrigla japonica, Solenocera crassicornis, Metapenaeopsis palmensis, Parapenaeus fissuroides* and *Calappa philargius*.

### Sample size and XGBoost

**Figure 3:**
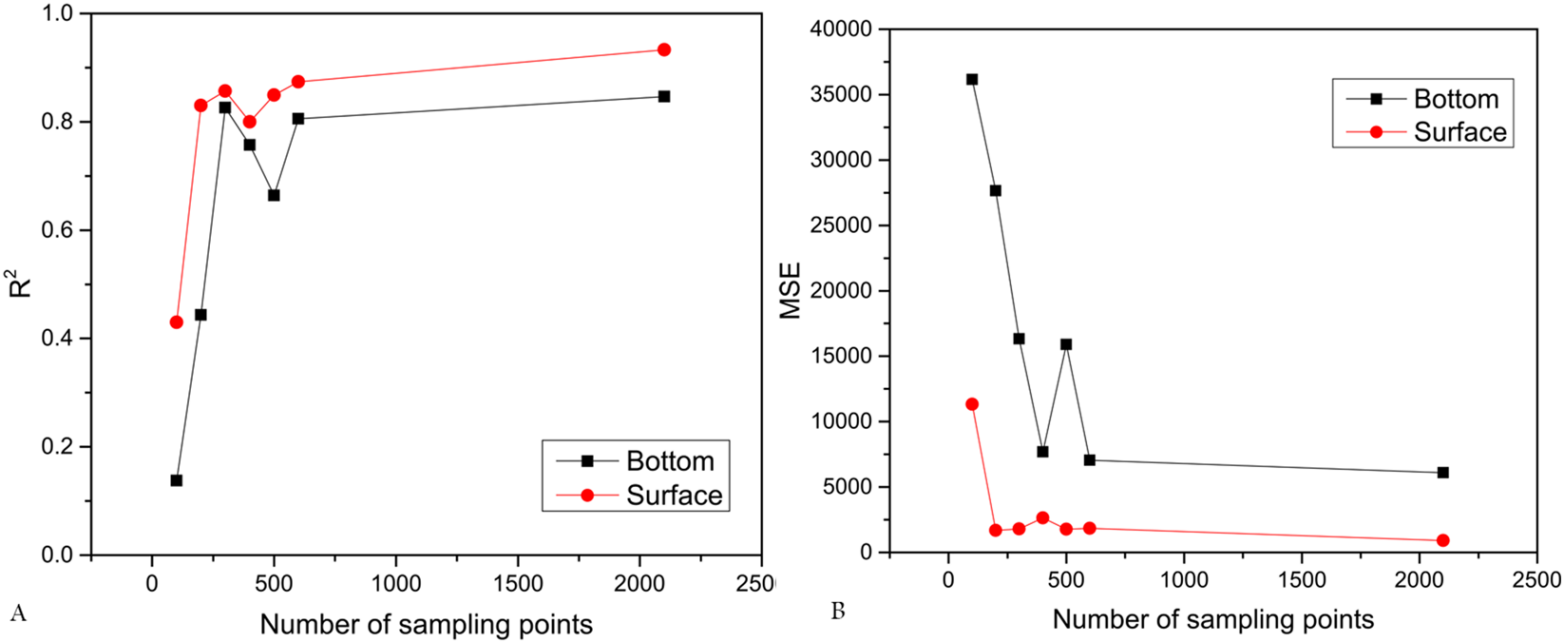
Relationships between goodness of fit (R^2^, **A**), mean square error (MSE, **B**) and the number of sampling points for 41 factors in the surface and bottom cold water layers using XGBoost.

The goodness of fit (R^2^) for XGBoost (default parameters) was the lowest (sample size = 100) before making adjustments. It increased with the increase in the sample size. It showed two peaks for surface and bottom NASCs when sample sizes were 300 and 600, and decreased until 400 for surface NASC and 500 for bottom NASC. At 2,100, the goodness of fit reached the highest value. The variation of MSE of the relationships was similar to R^2^, but some fluctuation was observed. Initially, MSE decreased rapidly, followed by a slight increase, and then reached the minimum (Fig. 3).

### Comparison of algorithms with the optimized sample size

**Figure 4:**
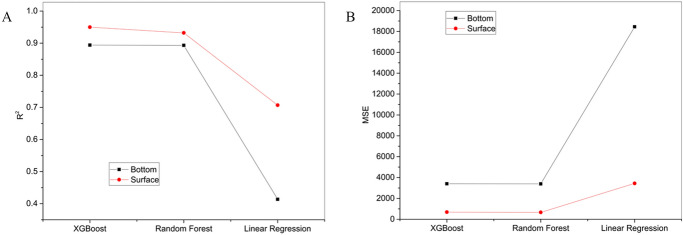
Comparison of goodness of fit (R^2^, **A**) and mean square error (MSE, **B**) of three models in different water layers (surface and bottom), the optimized sample size is 2,100.

XGBoost and random forests models showed similar performance, which was better than that of the linear regression model (Fig. 4). Both XGBoost and random forests models had good fitness for surface and bottom NASCs, whereas the linear regression model did not work very well. The fitness of the model for the surface mixed layer was higher than that for the bottom cold water layer. The difference of MSE and R^2^ from various models (XGBoost and random forests) was similar, and both models had low MSE.

### Factor sensitivity order

**Figure 5:**
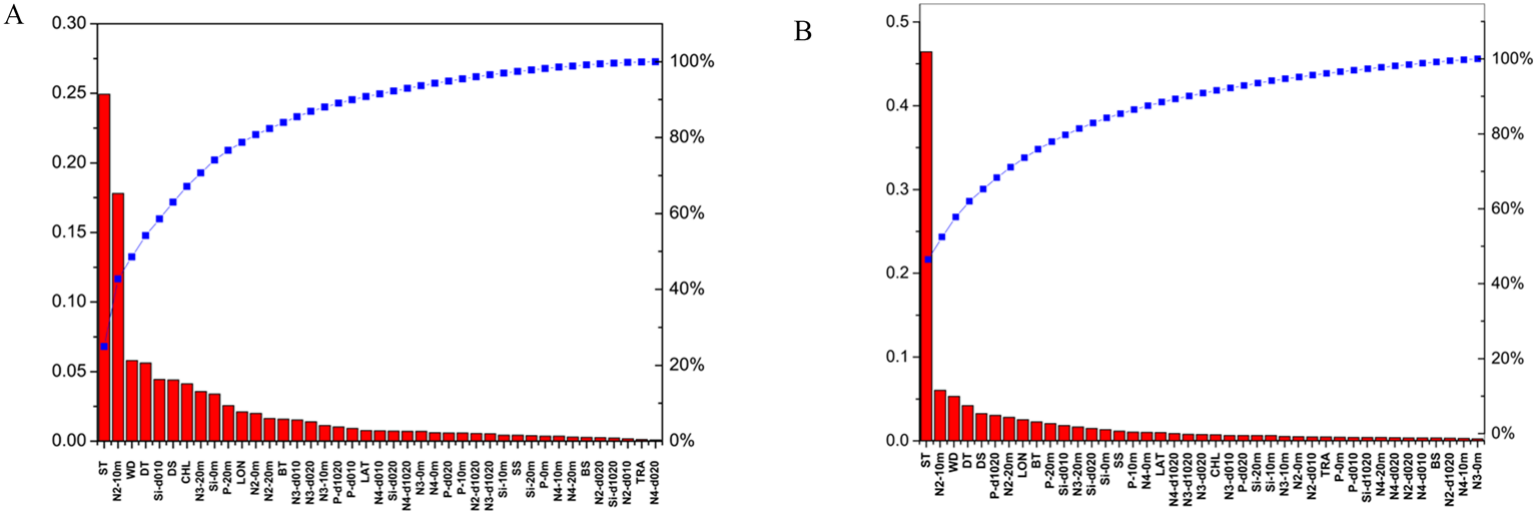
Sensitivity pre-sorting of 41 abiotic factors to surface fishery acoustic resource density using XGBoost (**A**) and Random forests (**B**).

The order of sensitivity of the factors considered in XGBoost and random forests models was similar, especially for the main relative and non-relative factors (Fig. 5). In XGBoost, some factors had significant sensitivity scores. In surface NASC, surface temperature (ST, °C) and NO_2_^−^ concentration at 10 m (N2-10 m, mg/L) had the highest sensitivity scores, which had a single contribution higher than 0.15, and the sum was 45%. Besides, WD, DT, Si-d010, DS, and CHL had some relatively minor importance, whereas BS and TRA contributed the least. In the random forests model, important factors were highly significant, with sensitivity scores higher than 0.45. ST was the most important factor for surface NASC, and BS and TRA had the smallest sensitivity scores.

**Figure 6:**
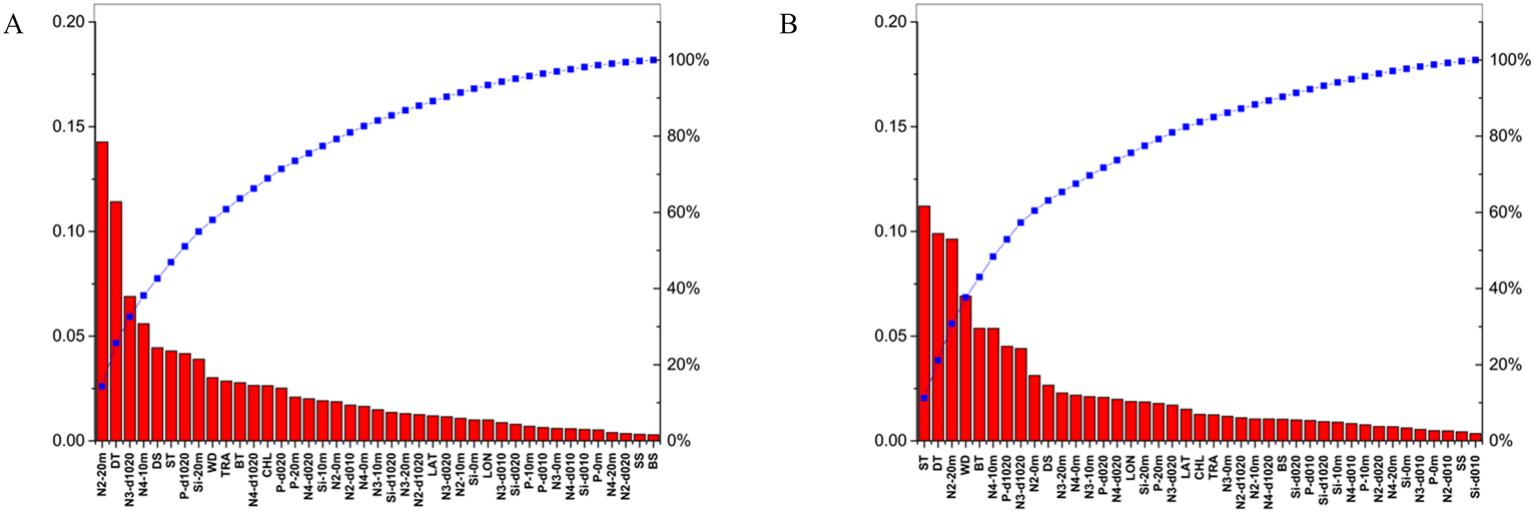
Sensitivity pre-sorting of 41 abiotic factors to bottom fishery acoustic resource density using XGBoost (**A**) and Random forests (**B**).

We observed differences in the sensitivity of factors in bottom obtained from XGBoost and random forests models. In XGBoost, the average contribution of each factor was lower than 0.15. For bottom NASC, N2-10 m and ST of the first featured factors had sensitivity scores higher than 0.1 and the sum contribution was 25%. Other factors made small sensitivity scores. In the random forests method, each factor had a contribution less than 0.12, and for bottom NASC, only ST of the first featured factors had a contribution higher than 0.1. DT and N2-0 m were also relatively important contributors (> 0.08). Other features had lower scores (< 0.08), such as WD, BT, N4-10 m, P-d1020, N3-d1020, N2-0 m, DS, etc., but most of them had non-zero contributions (Fig. 6).

### Surface and bottom factor sensitivity rank

**Figure 7:**
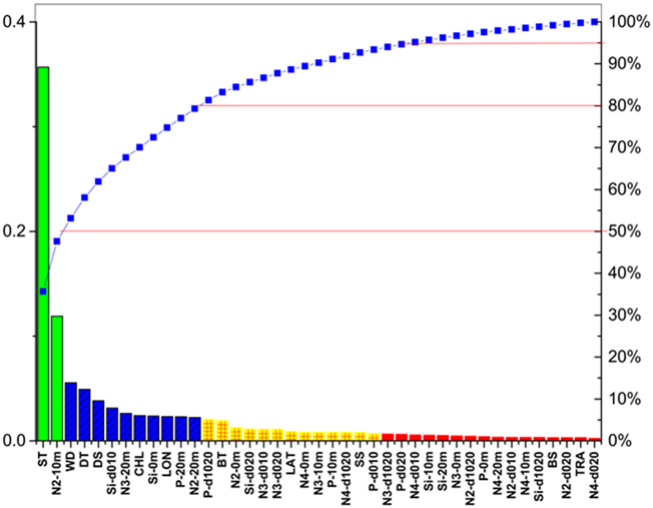
Sensitivity ranking of 41 abiotic factors to surface fisheries acoustic resource density using the integrated approach. Different colors denote the difference in sensitivity scores of factors. Green to red means level one to level four, set by the sum sensitivity scores (50%, 80%, and 95%, respectively).

The sum contribution of ST and N2-10 m, defined as first level-related factors for surface NASC within 20 m depth, was 50%, same as that of the left 39 factors (Fig. 7). ST had a great contribution of 36%, higher than 20%; this made it the most important factor influencing fishery resource distributions. Moreover, NO_2_^−^ at 10 m had a contribution of 12%, i.e., between 10 and 15%, making NO_2_^−^ at 10 m an important factor. The sum contribution of the second-level factors, mainly including WD, DT, Si-d010, N3-20 m, CHL, Si-0 m, LON, P-20 m, and N2-20 m, was about 30%. The sum contribution of the third-level factors was 15% that included 15 factors, namely, P-d1020, BT, N2-0 m, Si-d020, N3-d010, N3-d020, LAT, N4-0 m, N3-10 m, P-10 m, N4-d1020, SS, P-d010, N3-d1020, and P-d020. The sum contribution of the fourth-level factors was 5%, with 24 factors: N4-d010, Si-10 m, Si-20 m, N3-0 m, N2-d1020, P-0 m, N4-20 m, N2-d010, N4-10 m, Si-d1020, BS, N2-d020, TRA, and N4-d020 (Fig. 7).

**Figure 8:**
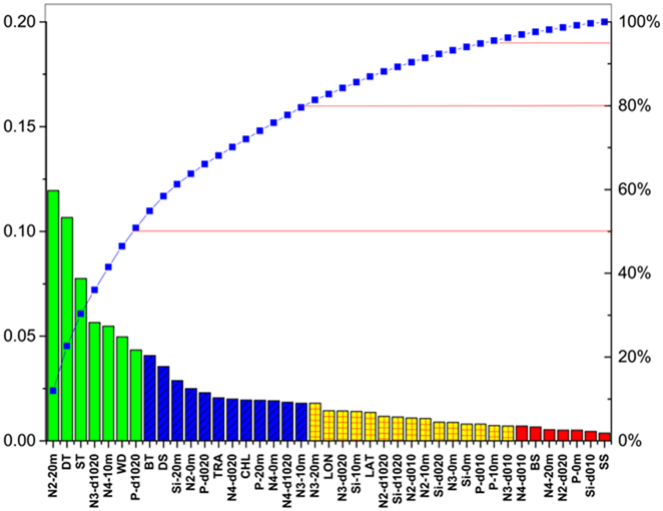
Sensitivity ranking of 41 abiotic factors to bottom fisheries acoustic resource density using the integrated approach. Different colors denote the difference in the sensitivity scores of factors. Green to red means the first level to the fourth level.

The sum contribution of the first-level related factors for bottom NASC within 20 m depth was 50%, which including N2-20 m, DT, ST (at surface mixed layer 2 m), N3-d1020, N4-10 m, WD, and P-d1020, same as the sum contribution of other 34 factors. The sum contribution of the second-level factors was 30%. There were 13 factors: BT (at bottom cold water layer 2 m), DS, Si-20 m, N2-0 m, P-d020, TRA, N4-d020, CHL, P-20 m, N4-0 m, N4-d1020, N3-10 m, and N3-20 m. The sum contribution of the third-level factors was 15%; it included 14 factors: LON, N3-d020, Si-10 m, LAT, N2-d1020, Si-d1020, N2-d010, N2-10 m, Si-d020, N3-0 m, Si-0 m, P-d010, P-10 m, and N3-d010. The sum contribution of the fourth-level factors was smallest with 5% only and included the following seven factors: N4-d010, BS, N4-20 m, N2-d020, P-0 m, Si-d010, and SS (at surface mixed layer 2 m; Fig. 8).

### Difference among the sensitivity of factors between surface and bottom cold water layers

**Figure 9:**
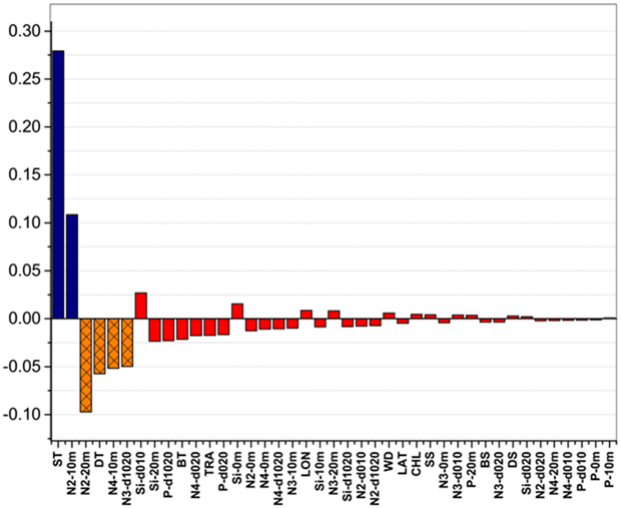
Difference in the sensitivity scores of the factors to the surface and bottom Nautical Area Scattering Coefficient (NASC). Dark blue: group A. Orange: group B. Red: group C.

We classified some factors as group A. They had considerably higher sensitivity scores to the surface mixed layer than to the bottom cold water layer, such as ST (2 m in the surface mixed layer) and N2-10 m, with a large positive difference. Some factors were defined as group B, which were contrary to group A with a large negative difference in the feature importance. These had higher sensitivity scores to the bottom cold water layer with a large negative difference, e.g., N2-20 m, DT, N4-10 m, and N3-d1020. The others were group C, which had low sensitivity for two water layers with a low difference (0 or smaller than 0.03), such as Si-d010, Si-20 m, P-d1020, BT, N4-d020, TRA, P-d020, Si-0 m, N2-0 m, and N4-0 m (Fig. 9).

**Figure 10:**
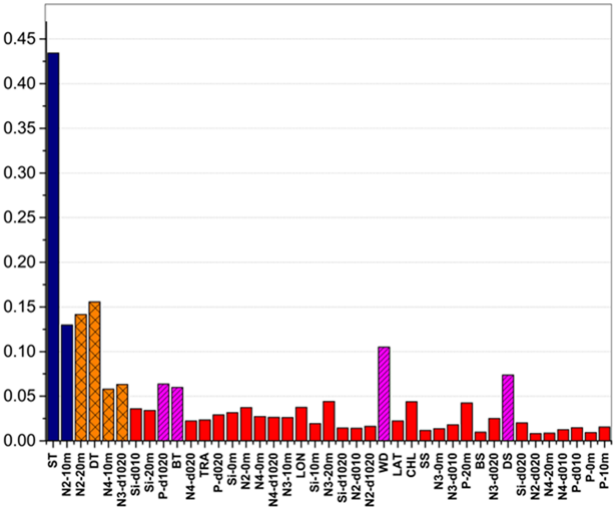
The sum sensitivity scores of factors to the surface and bottom Nautical Area Scattering Coefficient (NASC). Dark blue: group A. Orange: group B. Red: group C.

Factors belonging to group C, with a contribution higher than 0.05, were classified as group C + . They were important for both water layers and mainly included P-d1020, BT (2 m in the bottom cold water layer), WD, and DS. Other factors, with contribution smaller than 0.05, belonged to group C–, which were unimportant, such as Si-d010 and Si-20 m (Fig. 10).

## Discussion

### Algorithm and model performance

Both XGBoost and random forests models are machine learning algorithms with better performance (higher *R*^2^ and smaller MSE value) than the linear regression model under the condition that data quality and sample size are the same. However, the models did not yield good results when the data size was small. The model performance improved with an increase in the data size. Based on the interpolation methods, data size could be extended using coordinates^24–30^ so that the performance of XGBoost can be improved. Besides data size, the model performance could be improved by adjusting important parameters, such as time series periodic analysis with multiple data samples. The optimal model is able to make density predictions for both surface and bottom fishery densities under certain abiotic factors.

There are differences between XGBoost and random forests models^10,23^; thus, the sensitivity scores calculated by them are also different, especially for surface NASC, which had different contributions of different factors. The importance of features is different between the algorithms. Different algorithms resulted in different importance scores. The quantitative comparison in the form of scores can only be made while using the same algorithm. Nevertheless, the contribution of each factor was calculated in a similar way by all algorithms, especially for the factors with high sensitivity scores. Besides XGBoost and random forests models, support vector machine (SVM) ^31^ and logistic regression^32–34^ are available for feature selection.

### Contribution of the factors to surface and bottom NASC

It is supposed that NASC of different water layers is directly related to the factors of their own layer. For example, in the present study, surface NASC was related to ST and N2-10 m, which were first featured. Similarly, the bottom NASC was related to N2-20 m, which was the first level-related factor. However, special cases also existed. In the rank of the sensitivity of factors for surface NASC, certain surface factors, such as N2-0 m, N4-0 m, N3-10 m, SS (2 m above surface mixed layer), Si-10 m, N3-0 m, P-0 m, and N4-10 m, were less important than some bottom factors; all had smaller sensitivity scores than BT (2 m in the bottom cold water layer). In the rank of the sensitivity of factors for bottom NASC, BT and BS at 2 m of the surface mixed layer were less important than ST (2 m above the surface mixed layer). The possible reasons may be that the sensitivity of direct factors for water layers was smaller than that of other factors, such as food influenced by surface factors, or there may be no significant direct effects.

### Sensitivity scores of geographical, static, and dynamic factors to the surface and bottom NASC

The sum sensitivity scores of geographical, static, and dynamic factors to surface NASC were 0.087, 0.691, and 0.221, respectively, and average values were 0.029, 0.033, and 0.013, respectively. The results indicated that there were significant differences among the abiotic factors of surface NASC, and the sensitivity scores of static factors were higher than that of the dynamic and geographical factors, while dynamic factors were the weakest. Moreover, it showed that surface fishery resource density was more directly and highly affected by static factors than by other factors.

For bottom NASC, the sum sensitivity scores of geographical, static, and dynamic factors were 0.078, 0.530, and 0.392, respectively, and average values were 0.026, 0.025, and 0.023, respectively. Similarly, for bottom NASC, the sum sensitivity scores of static factors were the highest; however, the average value was close to the other two. It showed that the bottom fishery resources density was influenced by multiple factors. However, the human factors, such as overfishing, were not considered, and therefore we are unsure of its effect on the bottom fishery resources density.

### Important abiotic factors

We found that the factors had different contributions in different water layers. It could be the result of different compositions of fishery creatures. There could be some creatures in the quantity that were substantially affected by some factor or factors in the surface mixed layer, so that these factors would contribute highly to surface fishery density as the first level-related factors. Similarly, for the bottom cold water layer, it may have several creatures affected by different factors. Therefore, the bottom fishery resource density was the first level related factor for many species, which did not have significant factors influenced by multiple factors. There are many kinds of fisheries resources in the offshore of the Northern South China Sea, and the composition is complex. The majority of fishery creatures live in the bottom cold water layer.

Temperature is one of the major abiotic stress factors. ST above 2 m in the surface mixed layer, belonging to group A and level one, was the most important factor for both surface and bottom cold water layers. Moreover, it contributed the largest difference to fishery resources as compared with other factors. Sea surface temperature is one of the major factors influencing the surface layer. It has a direct impact on surface NASC, such as jellyfish that have a tendency for temperature and temperature difference^35^. However, it also had a great influence on the bottom NASC, probably because ST could indirectly affect the bottom cold water layer. For example, the temperature has an influence on fish parasites^36^ and fish community structure^37^. DT, belonging to the level two in group B, had an immense effect on bottom NASC, which was also one of the important dynamic factors in the first level, indicating that temperature change greatly influenced fish behavior^38,39^. However, the sensitivity and extent of the reaction to temperature variation differed with species and age^40^.

Nitrite is the intermediate oxidation state between ammonia and nitrate, and nitrite toxicity could affect fish. Nitrite is usually taken up across the gills along with chloride, which disturbs several physiological functions, including ion regulation, respiration, and cardiovascular, endocrine, and excretory processes^41^. There exists a large difference in nitrite toxicity among fishes based on multiple internal and external factors. Important factors include water quality (i.e., pH, temperature, and cation, anion, and oxygen concentrations), exposure time, species, size, age, and individual fish susceptibility^42^. N2-10 m, one of the important static factors for the surface mixed layer and belonging to level two in group A, directly affected surface NASC, which indicated that sea creatures are more sensitive to nitrite. N2-20 m was the first important feature in class B that had a direct impact on the bottom NASC, which belonged to one of the static characteristics of the near bottom. This also indicates that nitrite had a higher possibility of having a direct impact on marine life in the bottom layer. Besides, the factors related to nitrites, such as N3-d1020 and N4-10 m, only had also had some influence on the bottom cold water layer.

Water depth, belonging to group C, greatly influenced both surface and bottom NASC. The proportion of certain fish species increased with an increase in water depth. For example, the proportion of Cephalopods was relatively high within the range of 40 to 100 m, and the proportion of crustacean was higher within the range of 10–20 m^43^.

Salinity difference (DS), which belonged to group C and was one of the dynamic factors, immensely affected both surface and bottom NASC. Salinity varied slightly in the same period; therefore, SS (2 m above surface mixed layer) and BS (2 m above the bottom cold water layer) did not correlate with factors related to seasonal fish migration. However, DS still influenced the vertical distribution of both surface and bottom cold water layers.

In addition, P-d1020 and BT (2 m above the bottom cold water layer) had some effect on NASC. They may have an indirect effect on the distribution of fishery resources or a direct effect with a time lag, although there was no clear evidence of their significant sensitivity in this study.

On the contrary, there were certain factors with less influence on water layers, such as SS (2 m above surface mixed layer), BS (2 m above bottom cold water layer), P-0 m, N4-20 m, and N2-d020; however, it did not imply that they had no function. The spatial distribution and age structure of organisms vary within water layers, which could lead to differences in the sensitivity of factors for each layer. If the relationships between species and factors are certain, or the rank list of the sensitivity of factors could be acquired, then creatures and their proportion in different water layers could be estimated.

### Fishery resource distribution and other factors

There are many different kinds of abiotic factors, and only a few of them were used in this study. The abiotic factors collected at the same sampling point are concurrent. In fact, time-lagged data of some abiotic factors are also very worth studying, such as chlorophyll. Chlorophyll is often considered having a 30-day accumulation period prior to being reflected in higher trophic levels through ocean food chains^44,45^. However, the food chain is affected by many factors, e.g., human interference and alien species. Therefore, time-lag studies may be more suitable to be carried out without human interference. Similarly, synchronous studies are often susceptible to external factors, such as strong changes in the weather, which could lead to a big change in the sensitivity ranking of important factors by affecting surface mixed layer^46,47^. These may be related to the diverse behavior of marine organisms in the face of changing living conditions.

In addition to abiotic factors, the distribution of fishery resources may be affected by other ecological factors (human factors, biotic factors), especially bottom fishery resources. There may be many human factors that can affect the distribution of marine fishery resources^45^, including fishing, breeding, wastewater discharge, etc. The human factors affecting the seabed fishery resources described in the study mainly refer to the overfishing with bottom trawl as the main fishing method. Overfishing also affects the structure of the food chain, with unpredictable effects on time lag. As for biotic factors, different species act as biotic factors for each other, and their mutual relations include predation, competition, and symbiosis^48,49^. Further, even within the same species, there are intraspecific relationships.

Vertical probability distribution characteristics of fishery resources, obtained by fisheries acoustics techniques, are different from traditional fishing (i.e., bottom trawls and fishing nets with LED lights), which is featured with two dimensions. Here, the third dimension was added, making the analysis for fishery resources probability distribution more comprehensive and showing the importance of fishery resources density distribution in different water layers better. Stratification research on fishery resource density improved the evaluation of fishery resources. It was more multidimensional as compared with traditional plane analysis (e.g., fishery resources assessment model, physical habitat simulation model).
